# Serotonin signaling: a new player and therapeutic target beyond Long‐haul coronavirus disease

**DOI:** 10.1002/mco2.523

**Published:** 2024-03-31

**Authors:** Lan Bai, Fangfang Zhou, Long Zhang

**Affiliations:** ^1^ International Biomed‐X Research Center Second Affiliated Hospital of Zhejiang University School of Medicine Zhejiang University Hangzhou China; ^2^ Institutes of Biology and Medical Science Soochow University Suzhou China; ^3^ MOE Laboratory of Biosystems Homeostasis & Protection and Innovation Center for Cell Signaling Network, Life Sciences Institute Zhejiang University Hangzhou China; ^4^ Cancer Center Zhejiang University Hangzhou China

**Keywords:** COVID‐19, neurocognitive disorders, serotonin

## Abstract

During the coronavirus disease 2019 (COVID‐19) pandemic, a subset of individuals continues to suffer from symptoms including fatigue, post‐exertional malaise, dyspnea, bone loss, and memory and neurocognitive dysfunction for months and even years after infection. This clinical phenomenon has been labeled ‘Long‐haul COVID’ or ‘post‐acute sequelae of COVID‐19 (PASC)’; however, the underlying pathophysiological mechanisms remain unclear. In a recent study published in *Cell*, Wong et al. revealed that viral infection and type I interferon‐driven reduction of peripheral serotonin impaired hippocampal responses and short‐term memory through vagal neurons in patients with PASC. Therefore, the study provided novel insights into how serotonin links persistent viral inflammation with the neurocognitive symptoms of Long‐haul COVID and actionable therapeutic targets for patients with PASC.

## Graphical abstract

In patients with post‐acute sequelae of coronavirus disease 2019 or other post‐viral syndromes, persistent viral RNA and downstream chronic inflammation induce peripheral serotonin depletion through the type‐I interferon‐IFNAR‐STAT1 signaling pathway. Moreover, attenuated tryptophan absorption, impaired serotonin storage, and enhanced serotonin degradation are responsible for the reduction of serotonin. Systemic serotonin reduction, in turn, causes neurocognitive disorders by directly dampening vagal signaling.

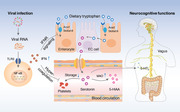

1

The ongoing coronavirus disease 2019 (COVID‐19) pandemic has brought about a plethora of unknown cellular and molecular changes in infected individuals. Wong et al. integrated published datasets and determined the metabolic alterations in patients with acute COVID‐19.^1^ Subsequently, they compared the plasma metabolomics of 58 patients who experienced post‐severe acute respiratory syndrome coronavirus 2 (post‐SARS‐CoV‐2) syndromes for more than three months with those of 60 patients infected with acute COVID‐19 and 30 who recovered. Among the altered metabolomics observed in these patients, serotonin levels were the most significantly decreased in patients with acute COVID‐19 and post‐acute sequelae of COVID‐19 (PASC). Moreover, serotonin levels could predict the development of PASC during the post‐acute phase, but not the acute phase, of COVID‐19 infection; thus, serotonin might be a valuable specific biomarker for PASC.

Serotonin was discovered in the 1930s, and its known functions, particularly in neuroregulation, have expanded over an unexpectedly wide range of areas. Serotonin reduction in patients with Long‐haul COVID may link SARS‐CoV‐2 with their persistent heterogeneous symptoms. Unexpectedly, the authors observed that plasma serotonin reduction was not a unique characteristic of COVID‐19 but was more general to systemic viral infection. This association has been verified in several mouse models of viral infection, including vesicular stomatitis virus (VSV), SARS‐CoV‐2, and its beta variant. Moreover, serotonin reduction in Long‐haul COVID could be attributed to persistent viral‐driven chronic inflammation in a mouse model of polyinosinic:polycytidylic acid (poly(I:C)) treatment. This finding raises the question of how viral infection‐driven chronic inflammation reduces serotonin levels. Notably, the authors found that the levels of type I interferon (IFN) and its related genes were significantly upregulated in both patients with Long‐haul COVID and mouse models, including those of viral infection and poly(I:C) treatment. Thus, the IFN response may trigger serotonin reduction. Indeed, blocking type I IFN signaling and genetic knockout of *Tlr3* or *Stat1* significantly prevented poly(I:C)‐induced serotonin reduction. Taken together, these results indicate that virus‐driven type I IFN inflammatory signals cause serotonin depletion (Figure 1).

**FIGURE 1:**
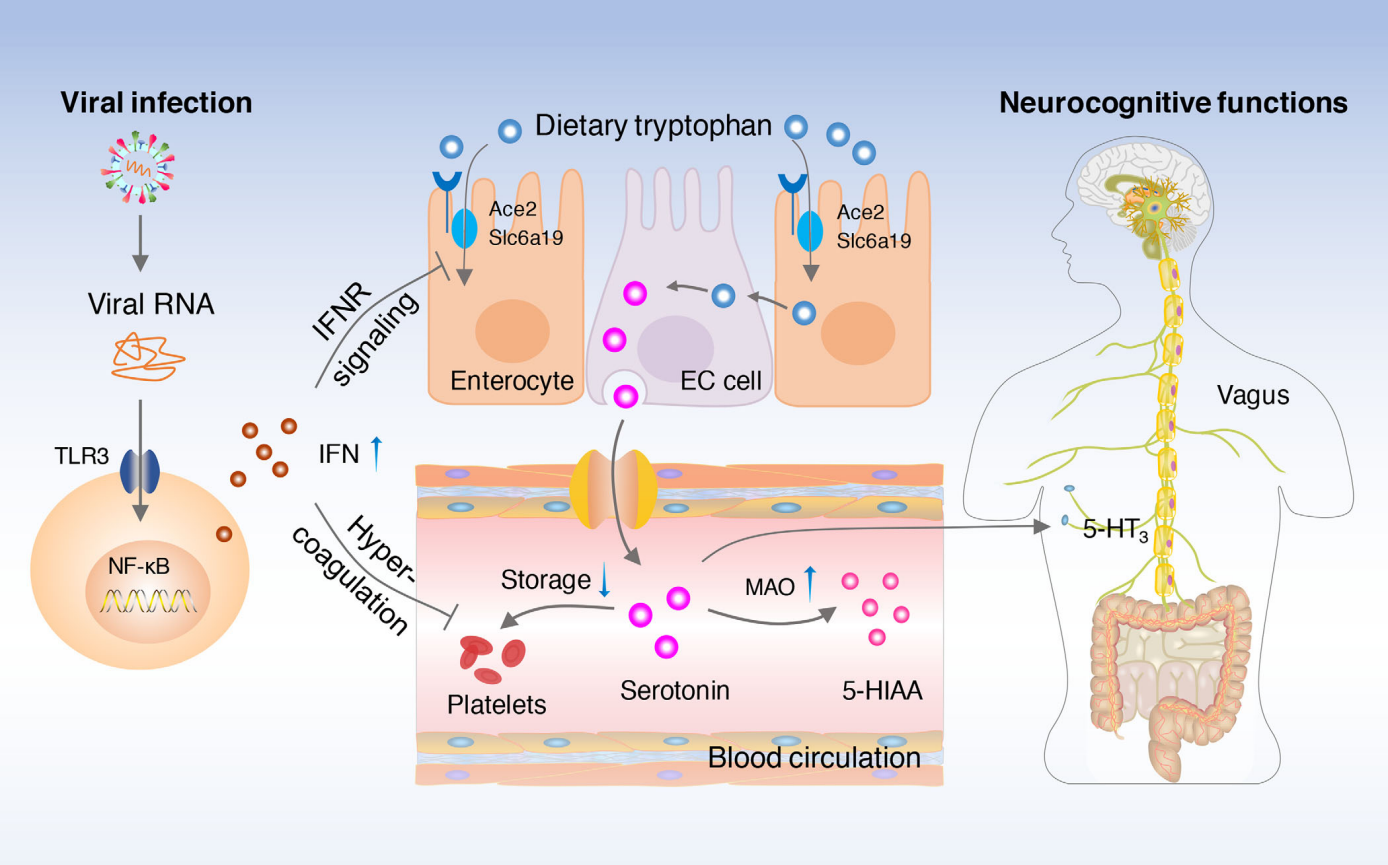
Viral RNA‐driven peripheral serotonin reduction impairs neurocognition by directly dampening vagal signaling. In patients with post‐acute sequelae of coronavirus disease 2019 (PASC) or other post‐viral syndromes, persistent viral RNA and downstream chronic inflammation induce peripheral serotonin depletion through the type‐I IFN‐IFNAR‐STAT1 signaling pathway. Moreover, attenuated tryptophan absorption, impaired serotonin storage, and enhanced serotonin degradation are responsible for the reduction of serotonin. Systemic serotonin reduction, in turn, causes neurocognitive disorders by directly dampening vagal signaling. Brain, gut, and blood vessel cartoon images were obtained from ScienceSlides. 5‐HIAA: 5‐hydroxyindoleacetic acid, 5‐HT_3_: serotonin receptor 5‐HT_3_, EC cell: enterochromaffin cell, IFN: interferon, IFNAR: interferon alpha receptor, MAO: monoamine oxidase, TLR3: toll‐like receptor 3.

Serotonin is produced primarily in enterochromaffin cells of the gastrointestinal epithelium and is released into circulation. The dynamics of circulating serotonin are influenced by several factors, including the availability of tryptophan, the primary dietary substrate for serotonin synthesis, as well as the biosynthesis, storage, and degradation of serotonin. In this regard, the authors investigated the significantly altered genes and signaling pathways in the small intestine of poly(I:C)‐treated mice and controls using RNA sequencing. Among these, the viral recognition and inflammation pathways were upregulated, and gene functions involved in amino acid absorption and metabolism were downregulated, although the serotonin biosynthetic pathway was unaffected. Consistently, the transcriptional levels of *Ace2* and *Slc6a19*, two key genes involved in tryptophan uptake, were significantly decreased in both mouse and human organoids after viral infection. Conversely, *Tlr3* or *Stat1* deletion and Nuclear factor kappa B inhibition prevented the responses to viral infection and restored the levels of *Ace2* and *Slc6a19*. Moreover, the downregulation of these amino acid absorption genes was associated with the presence of viral RNA in the stools of patients with PASC. Therefore, persistent viral RNA‐driven inflammatory signaling attenuates intestinal tryptophan absorption.

Platelets are the main stores for circulating serotonin, and free serotonin is short‐lived and oxidized by monoamine oxidase enzymes.^1^ The authors observed that viral infection‐driven inflammation promoted platelet hyperactivation, resulting in thrombocytopenia and a consequent impaired serotonin storage in a type I IFN‐dependent manner. Moreover, the degradation of free serotonin is enhanced by upregulating the transcriptional level of the *Maoa* enzyme during viral infection. Taken together, these results demonstrate that persistent viral RNA and infection‐driven serotonin reduction can be attributed to attenuated tryptophan absorption, impaired serotonin storage, and enhanced serotonin degradation (Figure 1).

Next, Wong et al. systemically studied the mechanisms by which serotonin reduction induced neurocognitive disorders in mouse models.^1^ They found that cognitive performance was impaired in mice exposed to acute VSV and chronic lymphocytic choriomeningitis virus and in poly(I:C)‐treated mice in a TLR3‐ and type‐I IFN‐dependent manner. Accordingly, the activity of the sensory neurons was restrained by poly(I:C) treatment. Conversely, neurocognitive performance in poly(I:C)‐treated mice was rescued by stimulating sensory neurons with the TRPV1 agonist capsaicin and restoring systemic serotonin levels with 5‐HTP, glycine‐tryptophan, or the selective serotonin reuptake inhibitor fluoxetine. Importantly, activation of hippocampal neurons can be restored by stimulating *Pox2b* ^+^ vagal neurons, and cultured vagal neurons can rapidly respond to serotonin stimulation. Moreover, the serotonin receptor 5‐TH_3_ was highly expressed in vagal neurons, and the functions of hippocampal neurons can be normalized by a 5‐TH_3_ agonist. Taken together, these results demonstrate that viral RNA‐driven peripheral serotonin reduction impairs neurocognition by directly dampening vagal signaling and that enhancing serotonin signaling could be an effective therapeutic strategy for patients with post‐viral neurocognitive disorders.

In conclusion, this outstanding study by Wong et al. systemically investigated the metabolites and transcriptional alterations in patients with Long‐haul COVID.^1^ Briefly, they found that viral RNA‐driven serotonin reduction, which occurred in a TLR3‐, IFNAR‐, and STAT1‐dependent manner, was the most significantly perturbed metabolite associated with PASC and was attributed to reduced substrate absorption, impaired serotonin storage, and enhanced serotonin degradation. Systemic serotonin reduction, in turn, causes neurocognitive disorders by directly reducing vagal signaling (Figure 1).

Wong et al. focused on exploring the pathophysiological mechanisms underlying the neurocognitive dysfunction of patients with PASC. However, Long‐haul COVID and other post‐viral syndromes are highly heterogeneous. It has been shown that SARS‐CoV‐2 infection can induce inflammatory bone loss,^2^ and the cation channel Piezo1, an important sensor of fecal microbial single‐stranded RNA in enterochromaffin cells, is associated with systemic serotonin synthesis and bone homeostasis.^3^ Thus, whether serotonin reduction observed in PASC is attributed to the dynamics of gut microbiota and is responsible for SARS‐CoV‐2 inflammation‐driven bone disorder and other symptoms is not clear. Moreover, the platelet‐derived serotonin metabolite 5‐HIAA can promote neutrophil recruitment to sites of inflammation during bacterial infections,^4^ suggesting that serotonin and its metabolites may have immunomodulatory properties in SARS‐CoV‐2 and other viral infections. Furthermore, serotonin not only facilitates the recruitment of innate immune cells and cytokine release in acute inflammation but also influences adaptive immune cell functions through specific serotonin receptors. Recently, serotonylation was reported as a new player in CD8^+^ T cell metabolism and functions.^5^ Thus, serotonin reduction observed in Long‐haul COVID could be a possible explanation for the deficient immune system and persistent inflammation‐associated symptoms, and boosting serotonin signaling using drugs might be a new hope for patients experiencing post‐viral syndrome.

Undoubtedly, this study by Wong et al. demonstrated how serotonin signaling links persistent viral inflammation with the neurocognitive symptoms of Long‐haul COVID, providing actionable therapeutic targets for patients with PASC.

## AUTHOR CONTRIBUTIONS

L.B. and L.Z. wrote the manuscript and prepared the figure. F.Z. provided valuable discussion. All authors have read and approved the final manuscript.

## CONFLICT OF INTEREST STATEMENT

The authors declare no conflict of interest.

## ETHICS STATEMENT

Not applicable.

## Data Availability

Not applicable.
